# Reproductive success is energetically linked to foraging efficiency in Antarctic fur seals

**DOI:** 10.1371/journal.pone.0174001

**Published:** 2017-04-28

**Authors:** Tiphaine Jeanniard-du-Dot, Andrew W. Trites, John P. Y. Arnould, Christophe Guinet

**Affiliations:** 1 Marine Mammal Research Unit, Institute for the Oceans and Fisheries, University of British Columbia, Vancouver, BC, Canada; 2 Centre d’Etudes Biologiques de Chizé, CNRS, Villiers en Bois, France; 3 School of Life and Environmental Sciences, Deakin University, Burwood, VIC, Australia; Institute of Zoology, CHINA

## Abstract

The efficiency with which individuals extract energy from their environment defines their survival and reproductive success, and thus their selective contribution to the population. Individuals that forage more efficiently (i.e., when energy gained exceeds energy expended) are likely to be more successful at raising viable offspring than individuals that forage less efficiently. Our goal was to test this prediction in large long-lived mammals under free-ranging conditions. To do so, we equipped 20 lactating Antarctic fur seals (*Arctocephalus gazella*) breeding on Kerguelen Island in the Southern Ocean with tags that recorded GPS locations, depth and tri-axial acceleration to determine at-sea behaviours and detailed time-activity budgets during their foraging trips. We also simultaneously measured energy spent at sea using the doubly-labeled water (DLW) method, and estimated the energy acquired while foraging from 1) type and energy content of prey species present in scat remains, and 2) numbers of prey capture attempts determined from head acceleration. Finally, we followed the growth of 36 pups from birth until weaning (of which 20 were the offspring of our 20 tracked mothers), and used the relative differences in body mass of pups at weaning as an index of first year survival and thus the reproductive success of their mothers. Our results show that females with greater foraging efficiencies produced relatively bigger pups at weaning. These mothers achieved greater foraging efficiency by extracting more energy per minute of diving rather than by reducing energy expenditure. This strategy also resulted in the females spending less time diving and less time overall at sea, which allowed them to deliver higher quality milk to their pups, or allowed their pups to suckle more frequently, or both. The linkage we demonstrate between reproductive success and the quality of individuals as foragers provides an individual-based quantitative framework to investigate how changes in the availability and accessibility of prey can affect fitness of animals.

## Introduction

Optimal Foraging theory assumes that natural selection favours animals that forage more efficiently, with foraging efficiency defined as the ratio of energy gained to energy expended to acquire food [1–3]. This implies that energy gained in excess of maintenance requirements can be allocated to reproduction, survival and growth [4]. Consequently, individuals that maximise their energy return per unit of energy (and time) spent have more energy (and time) to allocate to reproduction over their lifetime and thus a greater fitness than less efficient conspecifics. Foraging efficiency thus ultimately shapes the dynamics of populations.

Empirically testing the Optimal Foraging theory requires knowing how much energy is spent foraging, the nutritional quality and quantity of resource ingested, and a concomitant measure of reproductive success of individuals. Studies with controlled energy gain and energy expended, or with species in captivity with ‘rapid’ reproductive rates have yielded findings consistent with the Optimal Foraging theory [5, 6]. However, validating the theory in the wild is more complicated because of the difficulty of simultaneously measuring the energy intake and output of free-ranging individuals, as well as their reproductive success. This is particularly true for marine mammals that are long-lived and inhabit environments where direct observation of foraging is impossible. Studies have investigated life history traits including reproductive rates in marine predators [7, 8], but have generally not linked them to foraging efficiency. Others have looked at foraging efficiency indices, but often assumed that these indices are linked to fitness without explicitly linking the two parameters [9]. There is therefore a need to link reproductive success with measures of foraging efficiency, which would allow predictions to be made about how the individual fitness and population trends of top predators are affected by changes in prey availability and foraging behaviours.

The energetic cost of foraging in free-ranging pinnipeds can be assessed using indirect metabolic techniques such as heart rates or doubly-labelled water [10, 11] or more recently by accelerometry [12]. In contrast, the energy gained while foraging has been traditionally measured by identifying prey species in spews, scats, or stomach contents [13–15] and estimating numbers consumed from changes in body water pool [16], or with stomach pills that measure the changes in temperature between the predator’s body and the cold prey ingested [17–19], both of which present challenges in wild otariids. Consequently, studies have tended to either report foraging effort but not gain [20, 21], or foraging gain but not effort [22, 23], or have used behavioural indices rather than quantitative measurements of foraging efficiency [23–25]. More recently, however, tri-axial accelerometers have given access to measures of prey capture attempts in free-ranging marine predators [26–28]. This technological innovation makes it thus possible to quantitatively estimate foraging efficiency of individual marine predators by combining cost of foraging through one of the techniques mentioned above, with gain of foraging from diet composition analyses and measure of prey capture attempts using accelerometers.

Antarctic fur seals (*Arctocephalus gazella*) give birth to a single pup once a year. Mothers then nurse their pups for 4 months during which time they alternate periods of foraging at sea to replenish reserves and fasting periods on land while nursing their pups. Allocation of energy to their pup dictates growth rate and mass at weaning, which is directly linked to survival of the pup during their first year at sea, the critical period in the life cycle of fur seals [29–31]. Consequently, growth rates and mass at weaning of pups can be used as indices of annual reproductive success of female fur seals. As central place foragers, mothers are also time-limited during their foraging trip by the fasting capacity of their pups and must trade-off the time they take to replenish their reserves with the nutritional needs of their pups. Thus, given the time constraints mothers face while feeding, the allocation of their time to different activities at sea will affect both energy expenditure and gain. It is consequently important to study foraging efficiency linked to reproduction success within the context of individual time-activity budgets.

Antarctic fur seals can employ a range of foraging strategies depending on environmental conditions and the distribution of prey patches [32]. One means of predicting how they will respond to rapid changes occurring in their habitat [33] is to quantify foraging efficiency within the context of Optimal Foraging theory and investigate how it varies depending on behavioural choices and strategies of individuals at sea. It will ultimately provide information on how it impacts fitness via reproductive success. We thus sought to test links between foraging strategies, foraging efficiencies and proxies of reproduction success on a wild population of pinnipeds (Antarctic fur seals). We thereby determined 1) whether foraging efficiency of individual fur seals could be quantitatively estimated, 2) how their foraging behaviours shaped their foraging efficiencies, and 3) whether foraging efficiency affected reproductive success as indicated by the body size of pups at weaning.

## Material and methods

### Data collection

All data were collected on 20 lactating Antarctic fur seal females with a confirmed sucking pup at Pointe Suzanne, Kerguelen Island (Southern Ocean, 49°26'S—70°26'E) during the breeding season (Jan-Feb 2012) under the ethical regulations approval of the French Polar Institute (IPEV) and the UBC Animal Care Committee (# A10-0364).

Study females were captured using a hoop net and were brought to a restraint board where they were anaesthetized with isoflurane gas, weighed (± 0.5 kg) and measured for length and axillary girth (± 0.5 cm), and where DLW procedures were performed (see S1 Appendix). They were then equipped with Daily Diary tags (DD, Wildlife Computers^TM^, Redmond, WA, USA) that recorded tri-axial acceleration at 16 Hz and depth at 1 Hz (among other parameters), as well as Fastloc^®^ GPS MK10 loggers (Wildlife Computers^TM^) that recorded GPS coordinates, in addition to depth and water temperature at 1Hz. Both loggers were glued to the dorsal mid-line fur using a 2-part Devcon 5min epoxy glue. Finally, Gulf Coast Data Concept (GCDC) X6 or X8 accelerometers were glued on the head of the animals and recorded tri-axial acceleration at 16 Hz. Once the devices were securely attached and after the doubly-labelled water (DLW) metabolic measurements were completed (~ 2 h equilibration time), the females were released upon full recovery from the anaesthesia and allowed to rejoin the colony. Individuals were recaptured after a single foraging trip at sea and anaesthetized as previously described. The data loggers were removed by cutting the fur beneath the devices and a second set of morphometric measurements were taken. All methods for collection, analyses and calculations of the energy expenditure of female fur seals using the doubly-labelled water (DLW) are detailed in [34] and can be found in S1 Appendix.

### Foraging behaviours

We used depth data recorded by the DD tags to determine diving behaviours, or depth data recorded by the fastloc^®^ MK10 during the times that the DD tags malfunctioned. Time spent foraging or diving was calculated from time when the animal was below the water surface and performing confirmed dives (deeper than 3 m for more than 4 s) plus the post-dive interval as calculated from the Bout-Ending Criterion using the R package DiveMove [35]. The animals were considered to be transiting (i.e., traveling fast between 2 locations) whenever they were not diving and when the calculated speed at the surface (i.e., time needed to travel a distance between 2 GPS points) was > 1 ms^-1^. Distances traveled at the surface of the ocean (or horizontal distances) were calculated by measuring the linear distance between 2 successive GPS locations taking into account the curvature of the Earth using the Haversine formula. More details can be found in [34].

Average dive parameters, such as dive depths or dive durations, were nested within animals and were calculated using linear mixed-effect models with no fixed effects (only the intercept was calculated) and with individual as a random effect to take into account that each animal performed a different number of dives.

### Prey capture attempts

Prey capture attempts (PrCA) were measured using acceleration data while diving on the heave (z) and surge (x) acceleration channels from the head of the animals at 16 Hz [28]. We filtered the raw acceleration for these 2 channels using a 3^rd^ order high-pass filter at 3 Hz [28, 36] to obtain the dynamic acceleration signal from rapid head movements. These dynamic accelerations were then summed and a running variance was applied over a 2 s window. A cluster analysis on the resulting variance of dynamic acceleration was then performed using the k-mean function in R (2 clusters), which provided each animal with an individual threshold above which the signal was considered to correspond to a PrCA. Events detected within < 1 s of each other were considered coming from the same PrCA event (as assessed from feeding trials with live fish on harbour seals, A. Thomas, pers. com, and from video recordings of Steller sea lions [28]).

We tested the accuracy of acceleration from the back of the animals to detect PrCA by performing the same analyses as mentioned above but on the data collected from the DD tag and by comparing it to the results obtained from the head signals. Results show that back acceleration estimated PrCA as well as head acceleration does, with a slight overestimation of ~ 34 PrCA per night (S1 Fig, linear regression: PrCA_Back_ = 34. 25 + 1.00 × PrCA_Head_; *p* < 10^−15^, R^2^ = 0.90). Similar results have been found for head and back accelerometers deployed on southern elephant seals (C. Guinet, pers. comm.). Consequently, we calculated PrCA using back acceleration whenever the head accelerometer failed to record data over the full foraging trip.

Differences between prey capture attempts per day or per dive were estimated using linear mixed effect models with no fixed effect and with individual as a random effect to account for each animal performing a different number of dives or days at sea.

### Diet estimates

We determined diet composition of Antarctic fur seals from 20 scats collected at the Pointe Suzanne rookery during summer 2012 and from previously published values [37]. Samples were kept frozen at -20°C until ready to be processed in the lab following a standard procedure [38]. Hard part remains were identified to the smallest taxon possible following recommendations from [39]. Frequency of occurrence (FO) and relative proportion of each prey item in the diet were calculated using methods from [40] and compared to previously determined diet composition for Antarctic fur seals [32]. Upon identifying the main prey items, the size and energy content of the fish and squids found in the scats were taken from published sources [37, 39, 41–43]. The size and energy content of squids were averaged per year and then over the 3 years from [32] to obtain squid estimates, as most of the squid beaks we found were unidentifiable.

We obtained energy density of the diet (*ED*_*Diet*_) in g of fresh matter by averaging the energy density of different prey (*ED*_*i*_) weighted by their proportion within the diet (*P*_*i*_) over the number of prey in the diet *N* using:

$${ED}_{Diet}=\frac{\sum \left({ED}_{i}\times P_{i}\right)}{N}$$ (1)

Whenever information was missing for prey of low frequency of occurrence in the diet, we replaced it with the energy density of the closest related prey item or by the average of the energy content for the specific prey group. Once the mass (*BM* in g) and the energy density (*ED* in kJ/g) of each prey item (*i*) were estimated, we calculated the average energy content of a specific fish (*EC* in kJ) using:

$${EC}_{i}={BM}_{i}\times {ED}_{i}$$ (2)

The average energy content (*EC* in kJ) of a random non-specific prey (*p*) consumed by fur seals was calculated by weighting the energy content of a specific prey item by its relative proportion in the diet (*P*):

$${EC}_{p}=\sum_{i}\left({EC}_{i}\times P_{i}\right)$$ (3)

Means ± SD of energy content of each prey (*EC*_*i*_ in kJ) were calculated by generating 1000 values of mass and 1000 values of energy density (*ED*_*i*_) using normal distributions of their respective means ± SD (from Table 1). We calculated the error around *P*_*i*_ by bootstrapping scats (i.e., random sampling with replacement of individuals scats), and recalculating FO and SSFO for each new generated dataset (n = 1000). We then obtained the 95% CI and the SD from these values. Means ± SD of energy densities (*ED*_*Diet*_), and energy content of an average prey (*EC*_*p*_) in the diet were calculated by generating values of *EC*_*i*_ and *ED*_*i*_ for each prey type (*i*) in proportion to their respective importance in the diet (*P*_*i*_) out of 1000 values from normal distributions using their respective mean ± SD. For prey species with no *ED*_*i*_ or *EC*_*i*_ values, we used the average *ED* or *EC* from the prey group as their values weighted by their own proportion within the diet. As the prey group ‘Other’ did not have values for mass or energy density, we considered it as an average of the rest of the diet weighted by its relative importance in the diet.

**Table 1:** Relative proportion (%), average prey mass (in g), prey energy density (ED in kJ/g), energy content (in kJ) of prey groups in diets of female Antarctic fur seals breeding at Pointe Suzanne on Kerguelen Island. Groups ‘Other’ were assigned average diet values weighted by its percentage in the total diet for the calculation of *ED*_*Diet*_, and *EC*_*p*_. Bold values are for the total Prey group.

	Prey group	Perc.in diet (%)	Mass (g)	ED (kJ/g)	EC (kJ)
**Cephalopod**	Cephalopod	**12.11 ± 3.38**	**82.67 ± 32.05**	**4.05 ± 0.10**	**347.11 ± 4.14**
**Myctophid**		**75.50 ± 7.01**	**12.19 ± 0.11**	**8.56 ± 0.25**	**112.9 ± 0.94**
	*E*. *antarctica*	3.50 ± 1.50	3.20 ± 1.80	13.30 ± 2.60	40.97 ± 0.82
	*E*. *subaspera*	10.78 ± 1.66	11.80 ± 4.30	7.40 ± 1.00	88.45 ± 1.09
	*G*. *fraseri*	2.08 ± 1.11	5.17 ± 0.22	10.20 ± 3.50	52.63 ± 0.57
	*G*. *nicholsi*	9.11 ± 1.77	17.33 ± 1.95	9.80 ± 1.00	168.92 ± 0.84
	*G*. *piabilis*	14.11 ± 2.06	24.93 ± 0.87	9.50 ± 1.70	235.25 ± 1.36
	*G*. *sp*.	9.53 ± 1.88	15.81 ± 0.62	9.83 ± 0.90	155.48 ± 0.50
	*K*. *anderssoni*	0.83 ± 0.55	0.47 ± 0.12	8.10 ± 0.30	3.82 ± 0.03
	*P*. *bolini*	3.75 ± 0.85	0.87 ± 0.03	5.93 ± 0.38	5.15 ± 0.01
	*P*. *choriodon*	1.83 ± 2.27	0.87 ± 0.03	6.08 ± 0.55	5.28 ± 0.01
	*P*. *tenisoni*	12.11 ± 1.85	0.77 ± 0.20	6.23 ± 0.12	4.75 ± 0.04
	*Myctophidae sp*.	7.86 ± 1.52	NA	NA	NA
**Nototheniid**	*Nototheniid*	**4.44 ± 2.59**	**58.40 ± 0.00**	**5.03 ± 0.17**	**293.99 ± 0.31**
**Other**		**7.94 ± 2.54**	NA	NA	NA
	*S*. *hamiltoni*	1.25 ± 0.95	NA	NA	NA
	crustacean	3.33 ± 2.5	NA	NA	NA
	mollusc	2.36 ± 1.16	NA	NA	NA
	penguin	0.69	NA	NA	NA

### Foraging efficiency

The foraging efficiency (*FE*) of each seal (*i*) was calculated as the ratio between the energy expenditure at sea obtained from the DLW measures (*EE*_*i*_) per animal *i* and the energy gained while foraging at sea. Energy gained was estimated as the energy content of a non-specific prey (*EC*_*p*_) in their diet multiplied by the number of time seals *i* attempted to capture prey (*PrCA*_*i*_).

$${FE}_{i}=\frac{{EC}_{p}\times {PrCA}_{i}}{{EE}_{i}}$$ (4)

Seals with DLW results that were too close to background and seals that did not have acceleration data for the complete foraging trip were omitted from calculations. We are aware that PrCA represent attempts and not confirmed prey captures, but we assumed that unsuccessful PrCA were minor compared to successful ones (93% of attempts were successful in Australian fur seals [44], and that proportion of unsuccessful attempts were consistent between seals.

As the 3 parameters used to calculate the foraging efficiency of each individual animal (*FE*_*i*_) contain inherent errors, we calculated the resulting uncertainty around *FE*_*i*_ using the following 3 steps: **1)** error in *EE*_i_ was calculated by generating 1000 values following a normal distribution of 1.8 ± 7.2% of the measured values of DLW (error estimated by [45] when DLW was compared to respirometry on northern fur seals); **2)** error in *EC*_*p*_ was estimated by generating 1000 values using a normal distribution following the means ± SD for mass and energy density for each prey in the diet mentioned above; and **3)** error in *PrCA*_*i*_ was calculated by adding a detection error and subtracting a false positive error generated using uniform distribution between the ranges mentioned above to the measured *PrCA*_*i*_ value (1000 values generated). Detection rate of PrCA (true positive rate) is known to range from 68 to 97% (underestimation of true PrCA) and the false positive rate from 6 to 48% (overestimation of true PrCA) in Steller sea lions and Australian fur seals [28, 44]. Mean ± SD of *FE*_i_ was calculated over the 1000 generated *PrCA*_*i*,_ *EE*_*i*_ and *EC*_*p*_ using Eq 1. We calculated uncertainty over the average FE using the bootstrap method over 1000 simulations, where the random sampling with replacement were taken within the 1000 values of *FE*_i_ generated per animal in the study.

A sensitivity analysis was performed to estimate the contribution of each of the input variables uncertainty to the overall variance in the resulting foraging efficiency. This was done by computing the standardized regression coefficients (SRC), its bias and its 95% confidence intervals for each of the input variables using the *src* function in R (‘*sensitivity*’ library, R3.0.3) over 1000 simulated values.

### Pup growth

Thirty-six pups were randomly chosen at the Pointe Suzanne colony and were followed from birth until they could no longer be found on the colony. Mothers of 20 of the 36 pups initially followed were selected to be tracked. Standard morphometric measurements were recorded at birth and every 7–10 days or longer as the pups started to wander further from the colony. Length and girth were measured to the nearest 0.5 cm, and mass was recorded using scale at ± 0.1 kg.

Growth from birth to weaning of each individual pup was modeled with the von Bertalanffy equation [46] using the nls function (nlme package in R):

$$BM=A\times (1-e^{\left(-K\times age-t_{0}\right)})$$ (5)

where BM is body mass in kg, A is the asymptotic mass of pup at weaning (kg), *K* is the curvature parameter (d^-1^), age is the pup age in days and *T*_*0*_ is the age (in d) at which the pups have a mass equal to 0 kg. We also modeled the average male growth and the average female growth separately because male pups have a higher growth rate than female pups in fur seals [47, 48].

### Linking foraging efficiency of mothers and growth of pups

We calculated the difference between individual foraging efficiencies of each female and the average foraging efficiency of all the females as a metrics of relative quality of the mothers as foragers. We simultaneously calculated the individual theoretical mass at weaning (127 d) from individual pup growth curves and calculated the difference with the average mass at weaning calculated from the average sex-specific growth curve as a metrics of relative size at weaning of pups. We tested the relationship between these two metrics using a type II linear regression that took into account the fact that there were errors associated with both the response and the explanatory variables (*lmodel2* package in R) using the ranged major axis (RMA) method. We also tested the relative size of pups at weaning against other foraging metrics of mothers, such as time spent at sea or diving or rate of energy gain while diving using the same methods.

## Results

### Foraging behaviours and metabolic rates

**Fig 1:**
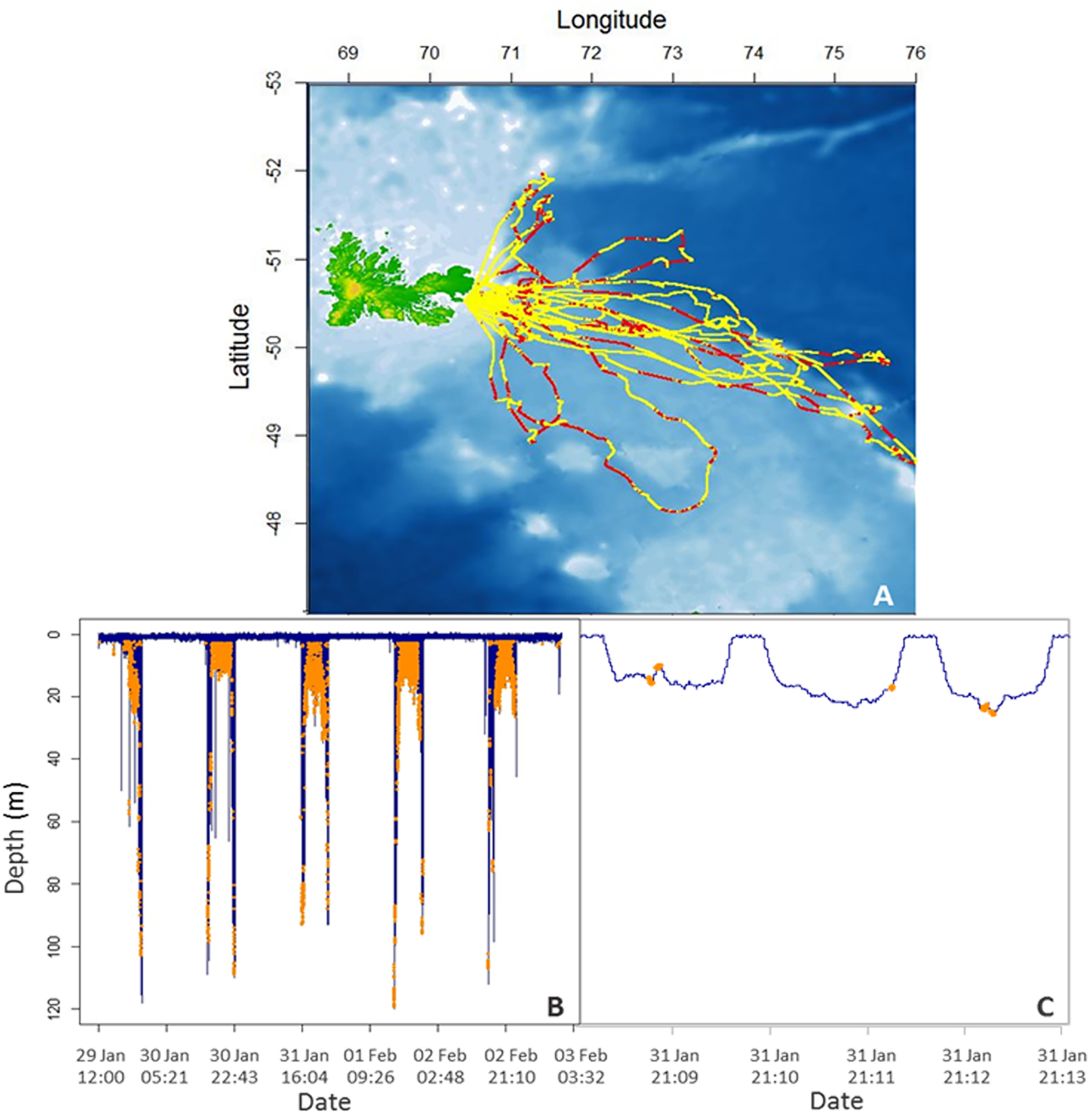
**Foraging locations of the 20 Antarctic fur seal females tracked on the Kerguelen plateau (A), and example of a dive profile during a foraging trip (B) or over a 5-min period (C).** Red dots show where the animals attempted to capture prey within the range of their foraging location (A, along the yellow GPS tracks) and orange dots where prey capture attempts occurred during the dives (B, C).

The female Antarctic fur seals all foraged east to south-east of Kerguelen Island on the Kerguelen plateau (Fig 1A). They weighed an average of 31.1 ± 0.9 kg prior to departure (range: 24.0–34.0) and gained an average of 0.6 ± 0.6 kg during their trips (2.2 ± 1.8% of their body mass). Their foraging trips were 635 ± 77 km long (range 271–1295 km) and lasted 7.6 ± 3.8 d on average (2.5–15.5 d). During these trips, they performed an average of 3949 ± 597 exclusively nocturnal dives at a mean depth of 19.9 ± 2.7 m (75.5% of which were less than 15 m deep) that lasted 42.6 ± 4.5 s on average. They spent 29.0 ± 0.7% (51.3 ± 5.9 h) of their time diving, 26.4 ± 1.6% (49.8 ± 7.9 h) transiting, 36.3 ± 2.0% (60.9 ± 7.6 h) performing surface activities, and 8.2 ± 1.7% (12.9 ± 3.0 h) resting.

Rates of energy expenditure per day at sea averaged 17.7 ± 1.1 MJ/d (0.59 ± 0.04 MJ/d/kg) for all females (n = 17; 3 of the original 20 females had isotopic levels too close to background levels for accurate measurements and were discarded). This translated into animals spending an average of 66.0 ± 7.5 MJ (2.2 ± 0.3 MJ/kg) while diving and 38.4 ± 6.4 MJ (1.3 ± 0.2 MJ/kg) while transiting from activity-specific metabolic rates [34].

### Prey capture attempts

Females attempted to capture prey 0.87 ± 0.11 times per dive while foraging (Fig 1B&1C). When only selecting dives in which at least one PrCA occurred, their capture rate increased to 2.04 ± 0.11 prey per capturing dive. This translated into females capturing an average of 336 ± 38 prey per night of foraging, and 2328 ± 387 prey over their entire foraging trip (range 704–6613 prey). When corrected for detection error as described above, the total PrCA over the foraging trip was slightly lower at 2139 ± 424 prey.

### Diet and prey energy contents

Diets of female Antarctic fur seals (Table 1) contained mostly myctophids (~ 75%), small mesopelagic fish of mass 1-25g with a high energy density (range ~ 6–13 kJ/g), and cephalopods (~12%) of bigger size (~83 g) but less energetically dense (~ 4 kJ/g). Overall, the energy content of their prey ranged from 5 to 350 kJ (Table 1). Given the contribution of each prey item to the total diet composition, the female fur seals ingested an average of 7.75 ± 2.47 kJ per gram of prey (*ED*_*Diet*_) with an energy content of 152.46 ± 1.08 kJ per average prey (*EC*_*P*_).

### Foraging efficiency and pup growth

Only 14 females out of the 20 we tracked had simultaneous data for energy expenditure (measurements were missing for 3 individuals, see above) and for prey capture attempts (accelerometer data were missing for 3 individuals) available to calculate foraging efficiency (Table 2). The calculated rates of energy gain for these 14 animals were 177.7 ± 21.4 kJ/dive, which was 130.6 ± 16.3 kJ/min spent diving and 37.6 ± 4.6 kJ per min spent at sea (54.1± 6.6 MJ/d). The average foraging efficiency for Antarctic fur seals was 3.44 ± 0.45 (range 1.24–6.86, 95% CI: 2.54–4.38). Sensitivity analyses showed that the largest contributor of the uncertainty around FE was related to diet estimates and to the estimate of mass of fish ingested. Uncertainty around PrCA estimates came second, and estimates of energy expenditure contributed the least to overall FE uncertainty (Table 3).

**Table 2:** Measured and corrected energy expenditure (EE in MJ), measured and corrected number or prey capture attempts (PrCA), energy gain (MJ) and foraging efficiency for female Antarctic fur seals during a foraging trip at sea, as well as mass at birth and at weaning for their respective pups (n = 14). The corrected values of EE and PrCA (calculated as explained in Foraging efficiency section of Material and Methods) were used with the estimated energy content per average prey (EC prey in kJ) to calculate the energy animals gained while at sea (in MJ) and their foraging efficiency (i.e., the ratio of energy gain/EE). The measured pup mass at birth, the calculated mass at weaning (from individual Von Bertalanffy growth models), and the deviation of individual pup mass at 127 from the average sex-specific mass at weaning (9.74kg for females and 11.73kg for males) of pups from the tracked mothers are indicated in kg.

Mom ID	Meas.EE (MJ)	Corr.EE (MJ)	Meas. PrCA	Corr. PrCA	Energy gain (MJ)	Foraging efficiency	Pup sex	Pup mass at birth (kg)	Pup mass at 127d(kg)	Deviation from average mass at 127d (kg)
21	229.31	225.20	7229	6613	1008.23 ± 168.09	4.50 ± 0.83	F	4.8	12.53	2.79
22	120.91	118.72	1976	1819	277.36 ± 45.11	2.35 ± 0.42	F	4.5	7.28	-2.46
23	46.50	45.70	1656	1519	231.63 ± 39.70	5.10 ± 0.96	M	5.1	14.27	2.54
26	246.67	242.49	4417	4082	622.28 ± 104.31	2.58 ± 0.47	M	4.7	10.69	-1.04
27	60.64	59.59	2093	1921	292.86 ± 49.79	4.94 ± 0.92	M	4.6	11.29	-0.44
28	50.06	49.17	768	704	107.32 ± 17.87	2.19 ± 0.40	M	4.6	13.66	1.93
29	35.88	35.25	1719	1576	240.33 ± 40.19	6.86 ± 1.27	F	4.6	12.29	2.55
31	46.09	45.26	1066	981	149.53 ± 24.92	3.32 ± 0.61	F	4.2	8.34	-1.4
32	59.00	57.90	1498	1364	207.97 ± 35.31	3.61 ± 0.67	F	5.0	11.08	1.34
33	112.36	110.24	1251	1148	174.95 ± 28.94	1.60 ± 0.29	F	4.5	8.76	-0.98
34	89.51	87.86	1410	1295	197.46 ± 33.31	2.26 ± 0.42	M	4.6	11.76	0.03
36	130.43	128.11	3844	3557	542.17 ± 92.39	4.26 ± 0.80	F	4.2	7.17	-2.57
37	193.83	190.14	2072	1909	291.04 ± 48.96	1.54 ± 0.29	M	4.9	7.18	-4.55
40	185.52	182.25	1600	1469	224.00 ± 38.24	1.24 ± 0.23	M	4.8	8.02	-3.71

**Table 3:** Standardized Regression coefficients (SRC), the min and max 95% confidence intervals, biases and standard errors (SE) of the sensitivity analysis on the calculated foraging efficiency of lactating Antarctic fur seals from energy expenditure at sea (EE in MJ), prey capture attempts (PrCA), mass (g), energy density (ED in kJ/g) and relative proportion in the diet of myctophids and cephalopods. We omitted prey groups with SRC below 0.1 in the Table.

Parameters	SRC	Min 95%CI	Max 95%CI	Bias	SE
**EE**	-0.576	-0.586	-0.564	0.000	0.006
**PrCA**	0.687	0.675	0.697	0.001	0.006
**Myct. Mass**	0.332	0.320	0.341	0.001	0.005
**Myct. ED**	0.103	0.094	0.111	0.001	0.004
**Myct. Prop.**	0.050	0.041	0.058	0.000	0.004
**Ceph. Mass**	0.221	0.211	0.229	0.000	0.005
**Ceph. ED**	0.145	0.135	0.154	-0.001	0.005
**Ceph. Prop.**	0.166	0.157	0.174	0.000	0.004

**Fig 2:**
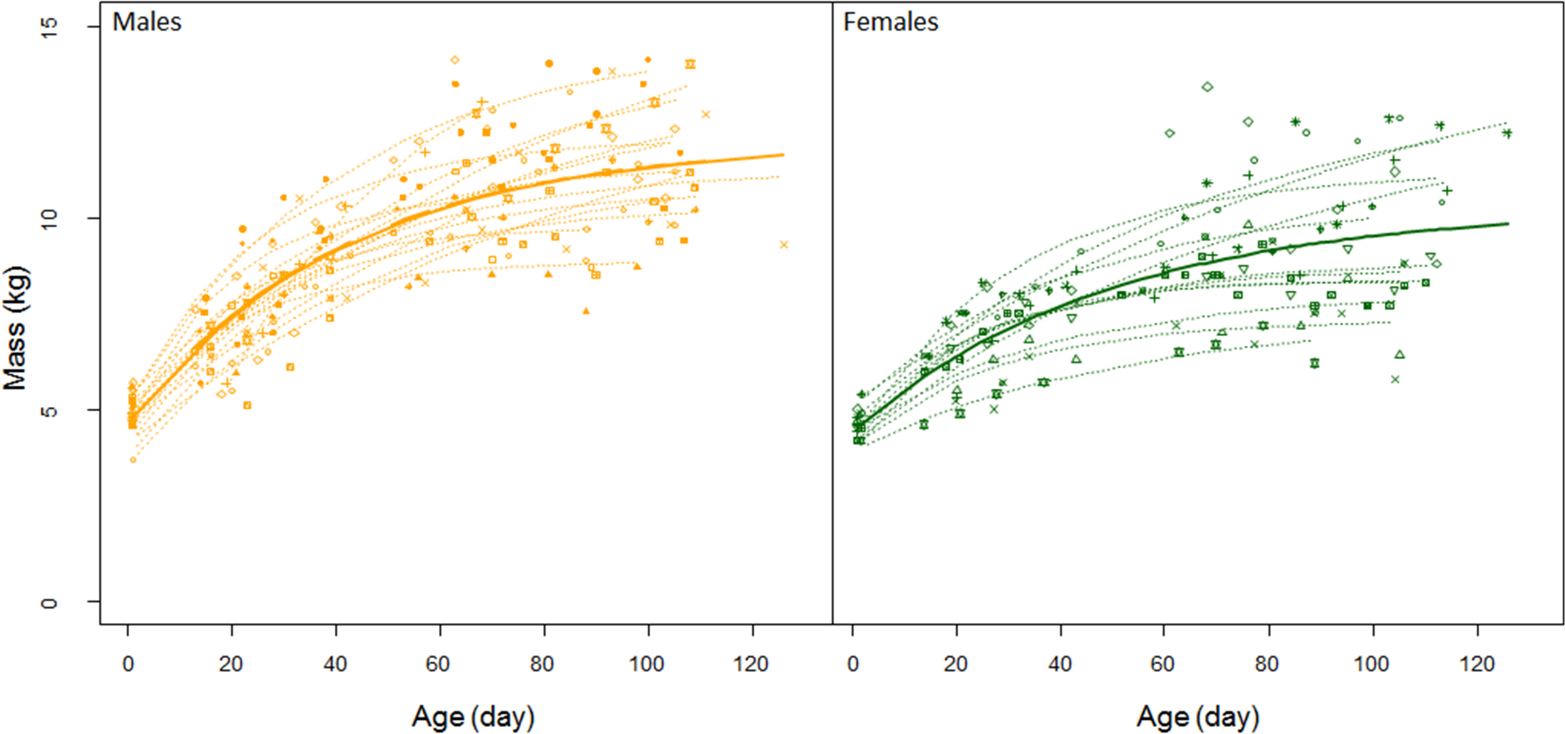
Growth of 36 individual Antarctic fur seal pups (n = 23 for males and n = 13 for females) from birth to weaning on Pointe Suzanne colony, Kerguelen Island in the breeding season 2012. Twenty of these pups belonged to mothers we tracked at sea. Dashed lines represent Von Bertalanffy growth curves fitted over individual pups during the nursing season and the solid lines are the average growth curve for all the pup of each sex. Plotting symbols are unique to individual pups).

Deviation from average foraging efficiency of individual females ranged from -1.85 to 3.56. Neither the mass, nor the body condition (estimated as the mass/length ratio [49]), nor the change in body mass of the females before and after foraging trips were linked to the foraging efficiencies of the females (all *p* > 0.62). Foraging trip duration or time spent performing different types of activities at sea were also not related to foraging efficiencies (all *p* > 0.21). Out of the 36 pups we monitored, 13 were females and 23 were males. Female pups weighed 4.6 ± 0.5 kg and male pups were 4.9 ± 0.5 kg at birth (*p* = 0.02). Three pups disappeared from our study site and we could not fit any growth curves to their data points, and 3 other pups had no data close to weaning which meant that the model could not determine an asymptote. Growth model for 17 male and 13 female pups (Fig 2) were:

$${BM}_{Male}=12.04\times (1-e^{\left(-0.038\times age+14.07\right)})$$

$${BM}_{Female}=10.26\times (1-e^{\left(-0.020\times age+27.65\right)})$$

average masses at weaning (127-d old) from these equations were 11.73 kg for male pups and 9.74 kg for females. Deviation from average sex-specific mass at weaning of individual pup mass ranged from +3.46 kg to -4.56 kg and averaged -0.31 ± 0.78 kg for females, and 1.09 ± 0.60 kg for males. Values for individual pups associated with tracked females for which foraging efficiencies are available are detailed in Table 2.

**Fig 3:**
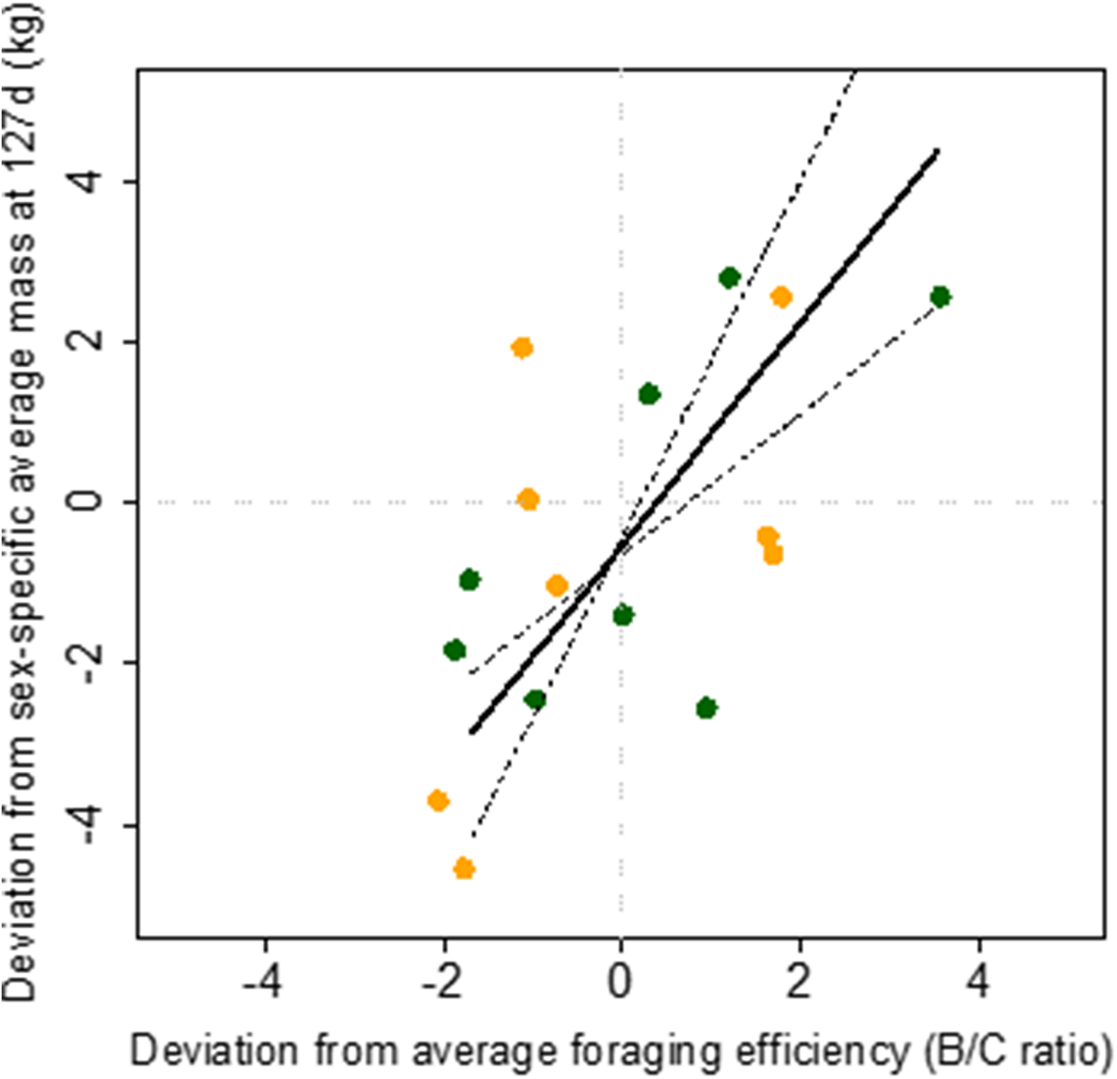
Deviation of individual Antarctic fur seal pup masses at weaning from sex-specific average pup mass at weaning as a function of the deviation of foraging efficiency of individual pup’s mothers from the average foraging efficiency over one foraging trip. Orange dots are male pups and green dots are female pups. Solid lines show the type II linear regression output (Y = 1.38 X—0.51, *p* = 0.0078, R^2^ = 0.41, n = 15. Spearman rank correlation rho = 0.62, *p* = 0.032).

**Fig 4:**
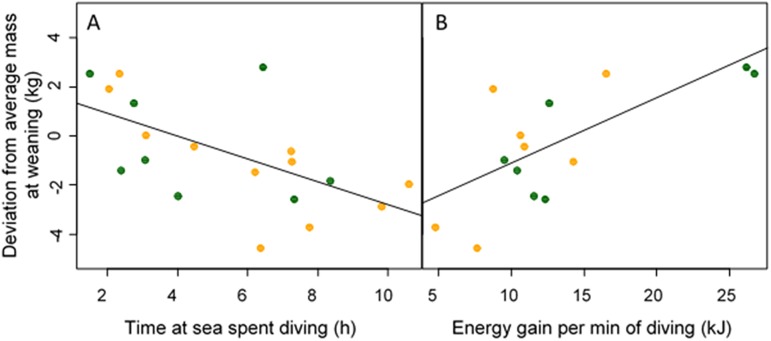
**Relationship between relative mass of Antarctic fur seal pups at weaning (calculated as deviation from sex-specific average pup mass at weaning) and time mothers spent diving during their foraging trip at sea in h (graph A, *p* = 0.0067, R**^**2**^ **= 0.36, slope = 0.046 ± 0.015, n = 19), and energy gain per min spent diving (Graph B, *p* = 0.0050, R**^**2**^ **= 0.49, slope = 0.026 ± 0.007, n = 14).** Time at sea was also linked to pup mass at weaning (*p* = 0.0166, R^2^ = 0.28).

The deviation of individual mothers foraging efficiency to average foraging efficiency was positively correlated to the deviation of individual pup mass to average mass at weaning (*p* = 0.0078, R^2^ = 0.41, Fig 3). The relationship in which the foraging efficiency of mothers was corrected for their size (mass-specific foraging efficiency) was also positive and significant (*p* = 0.02), but did not explain as much of the variation in the data (R^2^ = 0.29). Relative body condition of the pups (as expressed by the deviations compared to the sex-specific average mass at weaning), was however negatively correlated to the time females spent diving at sea (*p* = 0.0067, R^2^ = 0.36) and were positively related to the rate of energy gain per min of diving in kJ/min (*p* = 0.0050, R^2^ = 0.49, Fig 4). The relationship was also significant with foraging trip duration, but was not as tight (*p* = 0.0166, R^2^ = 0.28).

## Discussion

We calculated foraging efficiencies of individual female Antarctic fur seals from quantitative measures of at-sea energy expenditure and energy gained while foraging, and linked their foraging strategies to pup growth (an index of reproductive success). Foraging efficiencies were found to differ between individual fur seals irrespective of their time-activity budgets. In general, individuals attained high foraging efficiencies by increasing their rate of prey capture attempts rather than by decreasing the energy they expended while at sea. Pups whose mothers were relatively more efficient at foraging were bigger than average at weaning, which implies that pup growth rates reflected the quality of their mothers as foragers. This direct link between the foraging efficiencies of individuals and their reproductive success via maternal investment provides empirical support for the Optimal Foraging theory [3].

### Estimation of foraging efficiency (FE)

Our Antarctic fur seal females gained more energy than they spent during their foraging trips (all ratios > 1) with an average FE (gain/cost ratio) of 3.4 (~131 ± 16 kJ/min). Our results are consistent with foraging efficiencies calculated for free-ranging northern elephant seals (FE of 4), California sea lions (FE of 4 and energy gain of 112 kJ/min [16] and semi-captive Steller sea lions feeding on a simulated low prey-density patch (~ 206 kJ/min [50]). They are however much lower than the gain/cost ratio of 23 calculated for northern fur seals (406 kJ/min [16] or 20 for the same semi-captive Steller sea lions foraging on simulated high-density patches (~ 438 kJ/min [50]).

This 6-fold difference between different calculated foraging efficiencies shows that changes―or errors―in either costs or gains of foraging can greatly impact the final results. The doubly-labelled water method is known to be accurate at the population level, but to either over- or under-estimate metabolic rate at the individual level [45, 51]. Increase drag effect from animal-borne tags can also increase energy expenditure while potentially decreasing feeding success [52]. Furthermore, estimation of diet composition from scat hard-parts is also biased by differential digestion and retention rates depending on prey consumed and on meal sizes, and by the fact that it represents only the last 48 h before seals come back to land (where the scat is collected). It is also limited by the assumptions underlying the presence-absence methods of calculating relative proportion of prey in the diet [53, 54]. The degree of digestion of different hard parts also incorporate errors in the estimation of prey mass from size of fish otoliths, squid beaks or other hard parts [55, 56]. Variations in fish energy density by age, season and year [57, 58] can also contribute to error in calculated foraging efficiencies. Finally, validation studies of detection of prey capture attempts from acceleration on captive or free-ranging otariids using cameras simultaneously to accelerometers have shown that the accuracy of head acceleration to detect PrCA depends on animal behaviours, type of foraging (benthic versus pelagic) and on type and size of prey ingested [28, 44].

We applied correction factors when available, or included uncertainties in the final calculations, to account for the aforementioned biases as much as possible. The sensitivity analyses performed with the final estimates of foraging efficiency revealed that errors associated with PrCA affected the final result the most. This was not surprising given that different validation studies have not consistently agreed on their detection and false positive rates [28, 44]. Errors associated with mass of the prey affected FE more than energy density or relative proportion of prey in the diet, at least given the errors we estimated from the bootstrap method. Ultimately, all parameters related to foraging success (PrCA and diet-related parameters) tended to overestimate foraging efficiency, but this was partially compensated by the error associated with the doubly-labelled water method, which tended to underestimate foraging efficiency. Thus, it is not surprising that errors in foraging success affected the final foraging efficiency more than errors in cost given the larger number of parameters needed to calculate it. Consequently, particular care is needed to estimate parameters related to energy gain given the overall higher risk of error around foraging success compared to foraging expenditure.

### Foraging efficiency and pup growth

Despite all the sources of uncertainties listed above, foraging efficiencies of Antarctic fur seal females were positively related to the relative body size of their pups at weaning (Fig 3). Thus, foraging efficiency reflected maternal investment in the pup, or the extra energy available to allocate to reproduction (even though all energy available might not all be allocated to reproduction [59]). It is also important to remember that we measured foraging efficiency over a single foraging trip and that we compared it to overall growth of pups over the entire nursing season until weaning. Despite this, the fact that the relationship between quality of mothers as foragers and the pup size at weaning is significant suggests that 1) the relationship is extremely robust, and that 2) females were consistently good or poor foragers throughout the breeding season.

The mass of pups at weaning has been shown for a number of species to correlate to the pups’ chances of surviving the first year at sea [29–31], and thus by extension to the fitness of their mothers. Consequently, the robustness of the relationship between mother’s foraging efficiency and the growth of its pup shows that more efficient females at foraging have a greater reproduction success which is consistent with what the Optimal Foraging theory postulates. Fitness of an animal in its evolutionary sense should be assessed over its lifetime, but we only looked at the link between foraging efficiency and an index of reproductive success over a single reproduction cycle. We are aware that one reproduction cycle might not reflect lifetime fitness as individuals make trade-offs between reproduction and survival over their lifetime [7], but parents are usually consistent in their quality as foragers over years with few individuals producing a large portion of the next generations in top marine predators [60–62]. Applying these findings to our study implies that female fur seals that were better foragers during the breeding season would consistently produce bigger pups that would be better able to survive their first year at sea.

### Effect of time-activity budget

Flexibility in strategies reflects individual variability and the wide ranges of their adaptive behaviours to environmental conditions. Female fur seals typically display wide variation in foraging behaviours and time-activity budgets at sea [32, 63]. In our study, foraging trip duration ranged from 2.5–15.5 d, distance traveled 225–1295 km, and time spent diving or transiting from 22–34% or 15–43% of their foraging trip. Averages are within the ranges of previously reported values [25, 32, 64], but translate into a 7–8-fold difference between the minima of 15–16 h to the maxima of 105–125 h allocated to different activities between individuals.

Given the difference in metabolic rates associated with different types of activities [34], time-activity budgets would be expected to affect the foraging gain-cost ratio. However, we found no statistical relationships between foraging efficiency and time at sea or with any metrics of time-activity budgets, which indicates that quality of females depended more on individual capacities to extract energy from their environment rather than on time spent performing different activities at sea. The more efficient females in our study attained greater foraging efficiencies by having a greater rate of energy gain per min of dive time rather than by reducing their energy expenditure (which translated into rate of energy gained per min also being positively related to rate of pup growth). Females that were better at extracting food from their environment during dive time irrespective of energy costs produced bigger pups at weaning. This provides a direct quantitative linkage between the quality of females as foragers (i.e., at catching prey per unit of time spent diving), and their quality as mothers.

Females that spent less time at sea (or less time diving during their foraging trips) produced relatively bigger pups at weaning. Foraging trip duration is a common measure of foraging effort in fur seals [65–67]. In our study as in others [68], foraging trip duration was negatively related to relative pup mass at weaning, although the relationship was not as strong as with foraging efficiency, time spent diving, and rate of net energy gain while diving (all better indexes of pup growth rates). It is interesting to note that trip duration (or time spent diving at sea) and foraging efficiency (or rate of net energy gained while diving) were both linked to pup growth—but that trip duration or time diving were not related to foraging efficiency. This suggests that the two currencies that shape maternal investment in offspring (i.e., time and energy currencies) and thus pup growth might operate independently in some individuals.

It is difficult to tease apart whether females spending less time at sea produce bigger pups because they feed their pups more frequently, because they are more efficient foragers and return to land with greater energy overhead to allocate to feeding their pups, or both. In our case, foraging efficiency and feeding frequency (determined from trip duration) were both related to the size of pups at weaning, but foraging efficiency was a more accurate predictor. Marine mammals with a high maternal investment such as otariids are thought to optimize the frequency of feeding their offspring rather than their foraging efficiency to increase their success as reproducers [16, 69], but both could be confounding factors that relate to the quality of individuals in terms of efficiency at acquiring prey.

### Effect of phenotypic traits

Phenotypic traits that facilitate foraging efficiency should increase fitness of the animals if the efficiency with which mothers capture prey is the ultimate determinant of weaning mass and pup survival. Bigger females or females with better body conditions are thought to be better foragers, as they can dive aerobically for longer (have a higher ADL [70] and might be able to produce more milk through higher energy stores [71]. Foraging efficiency has been routinely estimated by measuring changes in body mass during foraging trip [9, 23, 64]. In our case, foraging efficiency was neither related to body mass, nor to changes in body mass, or changes in body condition indices. It is well known that female mass fluctuates during nursing bouts [64, 72], but we could neither control for, nor estimate, how long the females had been on land with their pups prior to capture. The fact that mass-related metrics did not relate to foraging efficiency could indicate different strategies in energy allocation between different essential physiological functions for females. Fur seals are income breeders, which means that they do not accumulate and store all the energy they need to provide their pups prior to the breeding season, but rather rely on energy obtained during frequent foraging trips within the nursing season [73]. In this case, animals have to determine energetic priorities between conflicting functions such as growth, maintenance, and reproduction during the breeding season itself. The uncoupling we observed between changes in body mass and foraging efficiency might indicate that some females compromised the growth of their pups to the benefit of their own physiological functions, or that some females might actually supplement the energy they acquire from foraging trips with limited body reserves, while others do not.

The lack of a relationship between the mass of females and their foraging efficiency is consistent with previous studies of Antarctic fur seals performed during years with favorable environmental conditions [23, 74], and indicates that 2012 was not a particularly challenging year for lactating fur seals. However, while maternal size does not contribute to difference in foraging efficiency between individuals in years of high food availability, it does positively influence pup growth rates during years of bad environmental conditions [23, 66]. This means that the physical advantages of larger females, probably also older and more experienced, makes a difference during years when environmental conditions are poor [23, 75] because accessibility of prey is likely to be more challenging and females are more likely to be foraging closer to their metabolic limits [69, 76]. A similar conclusion has been drawn for Adélie penguins [62] for which better foragers only held a reproductive advantage during challenging years. On the other hand, foraging costs become greater for individuals of larger size during years with normal conditions [77]. Consequently, the evolutionary pressure dictated by Optimal Foraging theory might select specific heritable phenotypic traits such as size, but the fact that these phenotypic traits might only become an advantage during years of challenging conditions could explain why there is so much variability in the population.

## Conclusions

The quantitative measures of maternal foraging efficiencies and offspring growth rates we found in free-ranging Antarctic fur seals provides empirical support that greater foraging efficiency of individual favors their reproduction success which is partly assumed in the Optimal Foraging theory. Direct energetic links between maternal investment and maternal foraging behaviours and efficiencies can help indirectly estimate the fitness of individuals and the dynamics of populations. Our findings further provide a quantitative energy-based framework to investigate and model the impacts of hypothetical and forecasted environmental and prey-related changes on the behaviours, and energetic costs and benefits of foraging by individual animals.

Antarctic fur seals, like all otariids, have an expensive reproductive system that can likely only be sustained in highly productive areas with concentrated and predictable high-energy content prey [7, 16]. Lactating females have been hypothesized to operate close to their metabolic ceiling [21, 76] which is consistent with the females in our study attaining higher foraging efficiencies by increasing their rate of energy gain rather than by decreasing their energy expenditure. This indicates that they might be physiologically and behaviourally limited in their capacities to adapt to drastic changes in environmental conditions.

## Supporting information

S1 Appendix Details on the methods used to measure metabolic rates with Doubly-Labeled Water in Antarctic fur seals. (DOCX)

S1 Fig Prey capture attempts (PrCA) by lactating Antarctic fur seals detected from back acceleration compared to PrCAs detected from head acceleration signals. **Each dot represents one animal.** The dotted line shows the results of regression model PrCA_Back_ = 34. 25 + 1.00 × PrCA_Head_ (R^2^ = 0.90, *p* slope < 10^−15^).
(TIF)
