# Supplementary material for: Reproductive success is energetically linked to foraging efficiency in Antarctic fur seals
Source: PLoS One. 2017 Apr 28;12(4):e0174001. doi: 10.1371/journal.pone.0174001 (PMC5409505; doi:10.1371/journal.pone.0174001)
Supplement: S1 Appendix — (DOCX) [file pone.0174001.s001.docx]

Measurements of daily energy expenditure (EE, kJ/day) were performed using the DLW method [[1](#_ENREF_1), [2](#_ENREF_2)]. While animals were under anesthesia, an initial blood sample was taken by venipuncture on the hind-flippers to determine ^2^H and ^18^O background levels. A known mass pulse-dose of DLW (622272 ppm ^18^O, 384645 ppm ^2^H) was then administered intravenously via a catheter on the other hind-flipper (0.3-0.6g/kg body mass) and flushed with saline solution to ensure full injection into the blood stream. Syringes were weighed before and after administration (± 0.0001 g, Sartorius balance) to calculate the mass of DLW injected. The labelled isotopes were allowed to equilibrate with the body water pool for 2 h during which the seals were either kept under very light anaesthesia or kept in a quiet closed environment. Equilibration times have previously been determined by serial blood sampling to be less than 2 h on fur seals [[3](#_ENREF_3), [4](#_ENREF_4)]. At the end of this period, a second blood sample was taken. A final blood sample was taken to determine isotope levels of ^2^H and ^18^O at the end of the foraging trip upon recapture.

All blood samples were collected in Monovette syringes (Sarstedt) coated with Li-Heparin and containing a plasma-red blood cells separator. Plasma was isolated from red blood cells by natural gravity separation for minimum 4 h as no electricity was available to power a centrifuge in the field. Plasma samples were then flame sealed into 2 x 100 µL glass capillary tubes, and stored at room temperature until isotopic analyses were performed. For isotopic analyses, plasma samples were vacuum distilled [[5](#_ENREF_5)] and the resulting distillate was used to produce CO_2_ and H_2_ [[methods in 6 for CO2](#_ENREF_6) , [and 7 for H2](#_ENREF_7)]. The isotope ratios ^18^O: ^16^O and ^2^H: ^1^H were analysed using gas source isotope ratio mass spectrometry (Optima, Micromass IRMS and Isochrom μG, Manchester, UK). Samples were run alongside three lab standards for each isotope (calibrated to International standards) to correct delta values to ppm. Isotope enrichments were converted to CO_2_ production for each individual using a two-pool model (i.e. considering respective individual dilution spaces for ^18^O and ^2^H), best suited for larger animals including pinnipeds [[8-10](#_ENREF_8)]. Initial isotope dilution spaces were calculated using the plateau method [[11](#_ENREF_11)]. We used the equation from [Speakman, Nair (12](#_ENREF_12)] to account for evaporative water loss when calculating metabolic rates from DLW concentrations [[13](#_ENREF_13)]. Finally, we converted CO_2_ production rates into daily energy expenditure using a respiratory quotient RQ of 0.80 [[9](#_ENREF_9), [14](#_ENREF_14)].

The study individuals spent time on land after the post-equilibration sample and upon return to the colony before they were recaptured and the final blood samples were collected. Energy spent during this ‘non-foraging’ time was part of the DLW measurement. Thus, we calculated energy expenditure at sea by subtracting on-land expenditure from the total estimate using previously determined values for lactating females in northern [[4.67 W/kg in 15](#_ENREF_15)] and Antarctic fur seals [[4.56 W/kg in 16](#_ENREF_16)] while on land.

Literature cited

1. Lifson N, McClintock R. Theory of use of the turnover rates of body water for measuring energy and material balance. J Theor Biol. 1966;12(1):46-74.

2. Butler PJ, Green JA, Boyd IL, Speakman JR. Measuring metabolic rate in the field: the pros and cons of the doubly labelled water and heart rate methods. Funct Ecol. 2004;18:168-83.

3. Costa DP. Isotopic methods for quantifying material and energy intake of free-ranging marine mammals. In: Huntley AC, Costa DP, Worthy GAJ, Castellini MA, editors. Approaches to marine mammal energetics. Lawrence, KA: Allen Press; 1987. p. 43-66.

4. Arnould JPY. Indices of body condition and body composition in female Antarctic fur seals (*Arctocephalus gazella*). Mar Mamm Sci. 1995;11(3):301-13.

5. Nagy KA. The doubly-labeled water (^3^HH^18^O) method: a guide to its use. Los Angeles, CA: University of California, 1983 Contract No.: 12-1417.

6. Speakman JR. Principles, Problems and a Paradox with the Measurement of Energy-Expenditure of Free-Living Subjects Using Doubly-Labeled Water. Stat Med. 1990;9(11):1365-80. PubMed PMID: ISI:A1990EG64900011.

7. Speakman JR, Krol B. Comparison of different approaches for the calculation of energy expenditure using doubly labeled water in a small mammal. Physiol Biochem Zool. 2005;78(4):650-67.

8. Schoeller DA. Measurement of energy expenditure in free-living humans by using doubly labeled water. J Nutr. 1988;118(11):1278-89.

9. Sparling CE, Thompson D, Fedak MA, Gallon SL, Speakman JR. Estimating field metabolic rates of pinnipeds: doubly labelled water gets the seal of approval. Funct Ecol. 2008;22(2):245-54.

10. Speakman JR. Calculation of CO_2_ production in doubly-labeled water studies. J Theor Biol. 1987;126(1):101-4. PubMed PMID: ISI:A1987H391400008.

11. Halliday D, Miller AG. Precise measurement of total body water using trace quantities of deuterium oxide. Biol Mass Spectrom. 1977;4(2):82-7.

12. Speakman JR, Nair KS, Goran MI. Revised equations for calculating CO_2_ production from doubly labeled water in humans. Am J Physiol. 1993;264(6 Pt 1):E912-7.

13. Visser HG, Schekkerman H. Validation of the doubly labeled water method in growing precocial birds: the importance of assumptions concerning evaporative water loss. Physiol Biochem Zool. 1999;72(6):740-9. doi: 10.1086/316713.

14. Dalton AJM, Rosen DAS, Trites AW. Season and time of day affect the ability of accelerometry and the doubly labeled water methods to measure energy expenditure in northern fur seals (*Callorhinus ursinus*). J Exp Mar Biol Ecol. 2014;452:125-36.

15. Gentry RL, Kooyman GL, editors. Fur seals - maternal strategies on land and at sea. Princeton, New Jersey: Princeton University Press; 1986.

16. Costa DP, Trillmich F. Mass changes and metabolism during the perinatal fast - A comparison between Antarctic (*Arctocephalus gazella*) and Galapagos fur seals (*Arctocephalus galapagoensis*). Physiol Zool. 1988;61(2):160-9.
