# Supplementary figures and images for: Reproductive success is energetically linked to foraging efficiency in Antarctic fur seals
Source: PLoS One. 2017 Apr 28;12(4):e0174001. doi: 10.1371/journal.pone.0174001 (PMC5409505; doi:10.1371/journal.pone.0174001)

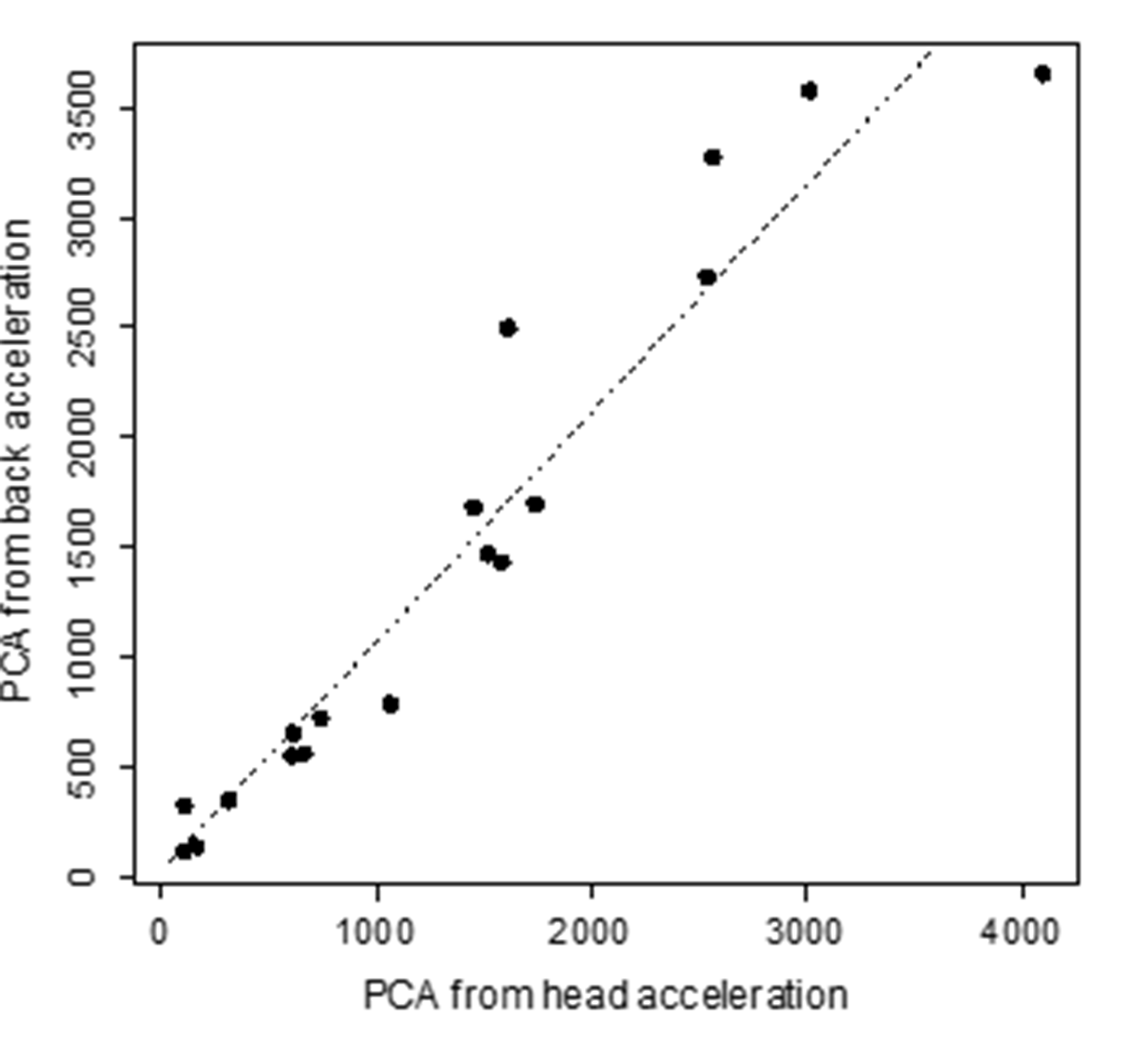

Supplement: S1 Fig — Each dot represents one animal. The dotted line shows the results of regression model PrCABack = 34. 25 + 1.00 × PrCAHead (R2 = 0.90, p slope < 10−15). (TIF) [file pone.0174001.s002.tif]
